# Hypersensitivity Reactions to Gadolinium‐Based Contrast Agents: Update From an Allergist's Point of View

**DOI:** 10.1002/clt2.70086

**Published:** 2025-07-28

**Authors:** Francesca Losa, Giovanni Paoletti, Giulia Costanzo, Fabio Lodi Rizzini, Marina Mauro, Donatella Preziosi, Federica Rivolta, Andrea Sangalli, Andrea Toniato, Serena Traversi, Alessandro Vrenna, Mario Di Gioacchino, Giorgio Walter Canonica, Enrico Heffler, Maria Teresa Costantino

**Affiliations:** ^1^ SC Allergologia Reumatologia e Immunologia Clinica Mantova Itlay; ^2^ Department of Biomedical Science Humanitas University Milan Italy; ^3^ Personalized Medicine Asthma and Allergy Humanitas Clinical and Research Center IRCCS Milan Italy; ^4^ Department of Public Health and Medical Science University of Cagliari Cagliari Italy; ^5^ DSCS (Dip. SCienze Cliniche e Sperimentali) ‐ Scuola di Specializzazione in Allergologia e Immunologia Clinica Brescia Italy; ^6^ Allergy Unit Division of Pulmonology S. Anna Hospital Como Italy; ^7^ Department of Internal Medicine Fondazione IRCCS Cà Granda Ospedale Maggiore Policlinico Milan Italy; ^8^ Allergy and Clinical Immunology Residency University of Milan Milan Italy; ^9^ Humanitas Gavazzeni e Castelli (BG) Ambulatorio di Allergologia Bergamo Italy; ^10^ Center for Advanced Studies and Technology G. D'Annunzio University Chieti Italy; ^11^ Institute of Clinical Immunotherapy and Advanced Biological Treatments Pescara Italy

**Keywords:** cross‐reactivity (CR), drug provocation tests (DPT), gadolinium‐based contrast agents (GBCAs), hypersensitivity reactions (HSRs), premedication, skin test (ST)

## Abstract

**Background:**

The increasing use of Magnetic Resonance Imaging (MRI) has led to a rise in the administration of gadolinium‐based contrast agents (GBCAs), accompanied by a growing number of reported adverse events (AEs).

**Objective:**

This review aims to provide an updated overview of hypersensitivity reactions (HSRs) to GBCAs, focusing on diagnostic and management strategies from an allergological perspective.

**Methods:**

We reviewed recent literature concerning the classification, clinical presentation, and pathophysiological mechanisms of HSRs to GBCAs. Particular attention was given to current recommendations for diagnosis, risk stratification, and prevention.

**Discussion:**

Adverse events to GBCAs are categorized into Type A reactions, which are dose‐dependent and predictable, and Type B reactions, which are dose‐independent hypersensitivity reactions. The latter may be allergic or non‐allergic, presenting diagnostic and therapeutic challenges.

**Conclusions:**

HSRs to GBCAs, though relatively rare, require careful evaluation and tailored management. An allergological work‐up, including skin testing and graded challenges when appropriate, plays a critical role in the safe re‐exposure of patients with prior reactions.

**Abbreviations**
AEs: AEs Adverse events
AGEP: Acute generalized exanthematous pustulosis
BAT: Basophil activation tests
CE‐MRI: CE‐MRI Contrast Enhanced MRI
CR: Cross‐reactivity
DPTs: Drug Provocation Tests
DRESS: Drug rash with eosinophilia and systemic symptoms
EMA: European Medicines Agency
EU: European Unit
GBCAs: GBCAs Gadolinium‐based contrast agents
GBFDE: Generalized bullous fixed drug eruption
ICD‐10‐CM: International Classification of Diseases, 10th Revision, Clinical Modification
ICM: Iodinated contrast media
IDTs: Intradermal Tests
IHRs: Immediate Hypersensitivity Reactions
IV: IV Intravenously
LTT: Lymphocyte transformation test
MRI: MRI Magnetic Resonance Imaging
NPV: Negative Predictive Value
PPV: Positive Predictive Value
PTs: Patch Tests
SCARs: Severe Cutaneous Adverse Reactions
SJS and 10: Stevens‐Johnson Syndrome and toxic epidermal necrolysis
STs: Skin Tests

## 1 Introduction

Contrast media for Magnetic Resonance Imaging (MRI) differ according to their effect on the electromagnetic field and their chemical composition. Paramagnetics are made with the metal gadolinium or manganese, most commonly gadolinium with an ion with a high magnetic susceptibility and the most stable ion with the highest number of unpaired electrons (GADO3+). It is the unpaired electrons that give the paramagnetic properties. Gadolinium derivatives can have a cyclic or linear structure and can be classified as ionic and non‐ionic [1]. To reduce the toxicity of metal ions, paramagnetic agents are administered in chelated form [2]. Since their introduction in the 1980s, they have been considered relatively safe compared to iodinated contrast media (ICM). Still, with their ever‐increasing use, the reporting of adverse reactions and hypersensitivity is increasing.

Adverse events (AEs) result not only from direct toxicity on the cells due to increased osmolarity but also from rare complications, such as nephrogenic systemic fibrosis due to the dissociation of gadolinium from the chelator to which it is bound [3] and neurotoxicity from potential deposition in brain tissue [4].

Hypersensitivity reactions (HSRs) are less common than toxic reactions and are predominantly immediate (≤ 6 h) or, more rarely, they are non‐immediate. The immediate reactions can be IgE or non‐IgE mediated. In the latter, the pathogenetic mechanism is supported by the degranulation of mast cells and basophils due to the direct action of the contrast medium on the cells, depending on the osmolarity and chemical structure of the contrast medium itself [5].

The data regarding allergy diagnostics related to the cross‐reactivity between the various molecules, the reliability of skin tests, and the schemes of provocation tests are limited. They are currently based on case reports and small case studies, which limit the interpretation of the results [6]. The role of premedication in patients with a generalized reaction to previous exposure to gadolinium contrast medium remains controversial, as it is not supported by solid evidence.

This narrative review aims to define the criteria for managing patients undergoing examination with GBCAs based on the most recent scientific literature.

## 2 Methods

A narrative review of the latest evidence regarding hypersensitivity reactions (HSRs) to GBCAs was conducted. The discussion was divided into six subsections, starting with the pharmacology of GBCAs. Next, it analyzed the epidemiological and clinical characteristics of HSRs to GBCAs. Finally, it provided an overview of the management of HSRs to GBCAs from an allergist's perspective, highlighting the pitfalls and unmet needs.

## 3 Discussion

### 3.1 Pharmacology of GBCAs

GBCAs are intravenously administered paramagnetic compounds used in contrast‐enhanced MRI (CE‐MRI). Their efficacy and potential toxicity largely depend on their structural and pharmacokinetic properties. GBCAs consist of a gadolinium ion (Gd^3+^), which is highly toxic in its free form due to its interference with calcium channels [7, 8, 9, 10], and a chelating agent that binds Gd^3+^ tightly to reduce tissue deposition and enhance elimination [11, 12, 13].

**FIGURE 1:**
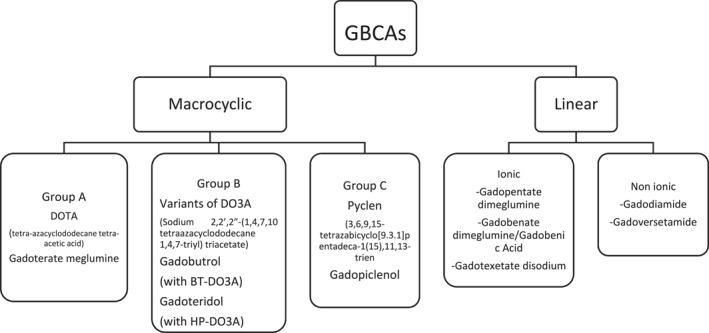
Classification of GBCAs based on strucutural class (macrocyclic vs. linear); each class is further subdivided according to ionicity (ionic vs. non‐ionic) and chemical structure of chelating agents (group A, B and C) (adapted from Gracia Bara et al. [1] and Vega et al. [14]).

GBCAs are classified as either linear or macrocyclic, depending on the structure of the chelating ligand (Figure 1). Macrocyclic agents form a cage‐like structure around the Gd^3+^ ion. They are generally more stable and less prone to transmetallation, the process by which Gd^3+^ is displaced by endogenous ions such as Zn^2+^, Cu^2+^, or Ca^2+^ [15]. These structural differences are clinically relevant, as macrocyclic agents are associated with lower retention and toxicity risks, leading regulatory agencies such as the EMA to restrict the use of linear agents [11, 13, 17].

Pharmacokinetically, most GBCAs are eliminated unchanged by the kidneys, with over 95% excreted within 12 h in individuals with normal renal function [11, 18]. However, in patients with impaired renal function (eGFR < 30 mL/min/1.73 m^2^), delayed clearance can increase the risk of gadolinium retention and adverse reactions [12, 19]. Notably, macrocyclic agents exhibit faster clearance and reduced deposition than linear GBCAs [18].

Although evidence of tissue deposition (e.g., brain, skin) exists, no clear clinical consequences have been demonstrated so far [19]. Nevertheless, the pharmacological properties of GBCAs—such as stability, molecular structure, and elimination kinetics—may influence the risk and type of hypersensitivity reactions, underscoring the importance of agent selection in patients with previous adverse events [6, 11, 17].

### 3.2 Epidemiology, Clinical Presentation and Risk Factors of Adverse Reactions to GBCAs

Since its introduction in the 1980s, GBCAs have been considered relatively safe compared to ICM. In recent years, due to their widespread use, several AEs were reported from allergic‐like HSRs to physiologic reactions [21, 22]. In late 2023, gadolinium‐specific diagnostic codes were added to the International Classification of Diseases, 10th Revision, Clinical Modification (ICD‐10‐CM) to describe gadolinium‐induced chronic toxicities as other complications like kidney injury, nephrogenic systemic fibrosis and encephalopathy [22].

The AEs frequency is low and ranges from 0.04% to 2.4%, with a true prevalence probably close to 0.1%, even rarer in children [1, 22]. Most AEs are predictable reactions (type A), probably caused by increased cell osmolarity and subsequent damage. Symptoms are generally mild: injection site reactions, thrombophlebitis, nausea and vomiting, headache, dizziness, paresthesias and arthromyalgias. Typically, they regress spontaneously. In some cases, it is difficult to distinguish anaphylaxis from vasovagal symptoms, especially when hypotension, bradycardia and sweating occur.

HSRs (type B) are less common, occurring from 0.004% to 0.7%. Immediate HSRs (IHSRs) represent the majority of HSRs (75%–100%); anaphylaxis counts for 0.01%, with a death rate of about 0.0019%. They occur within 1–6 h, caused by mediators released from mast cells and basophils [1]. In vitro studies ruled out that GBCAs are naturally histamine‐releasing drugs, so IHSRs might or may not be IgE‐mediated. The results from the in vivo test confirm the role of IgE in the pathogenetic mechanism of IHSRs. Still, the low positive predictive value of Skin Tests (ST) suggests alternative methods to activate mast cells and basophils (e.g., complement or MRGPRX2 receptor activation or bradykinin generation). An alternative recent hypothesis is that mast cells can be activated by meglumine, but not through the MRGPRX2 receptor. Non‐IgE‐mediated mechanisms could explain allergic‐like reactions at the first exposure [23]. IHSRs are mild in more than 90% of cases, and urticaria is the most common clinical manifestation. Non‐immediate HSRs (NIHSRs) occur from 1 hour to 1 week after GBCA exposure; they are more difficult to report, so they are probably underestimated [21, 22, 25]. They have an incidence of 0.05% and occur on the same administration day in about half of the cases. The commonest presentation is urticaria (66%), followed by maculopapular exanthema (33%) and itch (6.6%) [25]. Data about severe cutaneous adverse reactions (SCARs) are limited. Still, it is reported a drug rash with eosinophilia and systemic symptoms (DRESS) in a 13‐year‐old boy [26] and 2 case reports of acute generalized exanthematous pustulosis (AGEP); in all three cases, the culprit was gadobutrol. To date, in the literature, there are no cases of generalized bullous fixed drug eruption (GBFDE) and Stevens‐Johnson Syndrome and toxic epidermal necrolysis (SJS and 10) induced by GBCAs [27]. Post‐marketing safety data showed that all GBCAs can potentially induce HSRs, with the lowest probability for non‐ionic GBCAs and a tendency for more reactions for ionic macrocyclic GBCAs than linear ones [22, 29]. Gadobenate dimeglumine and gadoteridol showed more anaphylaxis/anaphylactoid shock reactions and gadoversetamide was more linked to SCARs [29, 30].

Nowadays, the leading risk factor for a GBCA HSR is a history of previous reactions. Considering ICM, it is unclear if an earlier AEs to another kind of contrast media is related to a higher risk of AEs toward GBCAs [6]. Suppose it is unlike a cross‐reactivity between ICM and GBCAs, especially regarding an IgE‐mediated mechanism. In that case, some authors suggest a possible role of an excipient, trometamol, present in both ICMs and GBCAs, but data are still lacking [30]. Female gender is also associated with a higher frequency of AEs related to GBCAs. This data is not surprising, considering that women have an increased incidence of AEs for many drugs. There could be both biological and socio‐economic reasons: certainly, the different hormonal balance plays a role in immunological response to drugs; on the other hand, women generally take more medications than men [32, 33]. The site of the scan could be correlated to AEs, in particular abdominal and thoracic examination, more than another site (brain or spine) [6].

However, taking an extensive patient's history is mandatory since chronic conditions like kidney or autoimmune diseases represent intrinsic risk factors for AEs [6].

### 3.3 In Vitro and In Vivo Diagnostics for GBCAs HSRs

HSRs to GBCAs are IHSRs or NIHSRs, the latter extremely rare. Accurate diagnosis of GBCA allergy is crucial to prevent future adverse events and guide clinical management.

After the reaction, an accurate clinical history is essential to record the GBCA administered and the interval between its administration and the onset of clinical symptoms.

For IHSRs, STs should be conducted within 2–6 months after the reaction, although no studies have analyzed the ideal timing for performing the tests [6].

STs must follow the EAACI‐ENDA recommendations using undiluted GBCAs for skin prick and dilution and 1:10 for intradermal tests (IDTs). Undiluted GBCAs can irritate intradermal testing and induce false positive results [34, 35]. In severe reactions, some authors recommend IDTs with 1:1000, 1:100, and finally 1:10 dilution [36, 37]. A panel of several GBCAs of different molecular structures and the culprit GBCA should be used for testing. STs' positivity increased with the severity of the reaction. The STs' negative predictive value (NPV) is between 80% and 90% [37]. So, it could mean that STs might provide a safe alternative to GBCAs, but it is not always possible to demonstrate IgE‐mediated reactions. Moreover, it is necessary to evaluate the cross‐reactivity (CR) risk, as described above. Until now, data regarding STs' positive predictive value (PPV) is still lacking.

Patch tests (PTs) with undiluted GBCAs are suggested for NIHSRs in case of negative IDTs at a 1:10 dilution with delayed readings, which are considered the most diagnostic tool. If negative, some authors performed IDTs with the undiluted GBCAs [39, 40].

Taking account of SCARs, allergological assessment could be challenging because in vivo tests have been considered a contraindication. Indeed, STs, both prick STs and IDs, carry on a risk of eliciting a reaction relapse. PTs are safer than STs and are the first choice; the timing to perform the test is at least 6 months after complete recovery of the disease. In case of negative PTs, STs and IDs with delayed readings are allowed for DRESS or AGEP, but not for GBFDE and SJS/TEN.

Generally, in vivo tests should be preferred to find safety alternatives to the culprit agents [27].

In vitro tests are available only in research settings, not in clinical practice. Measuring specific IgE antibodies to GBCAs can identify sensitization, but there is no in vitro demonstration of GBCA‐specific IgE [1]. Basophil activation tests (BAT) have gained prominence recently due to their sensitivity and specificity. Where available, it can help, especially in cases of severe reactions when STs or challenges are a risk to the patient or when these are contraindicated. BAT is considered positive if a drug induces the expression of CD203 C, an activation marker expressed on the basophils' surface. BAT was applied to GBCAs in a few cases. In the GBCAs, this diagnostic tool appears to be a complementary test, with a sensitivity of 68% and specificity of 93%. No data on applying the lymphocyte transformation test (LTT) in GBCAs HSRs [41, 42, 43].

### 3.4 Risk of Cross‐Reactivity Between GBCAs

**FIGURE 2:**
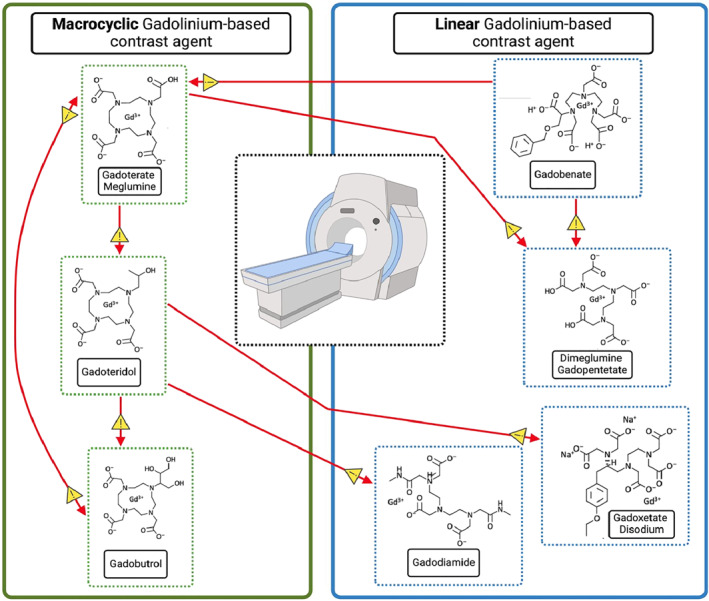
Cross‐reactivity patterns in IgE‐ and T‐cell‐mediated hypersensitivity reactions to gadolinium‐based contrast agents (GBCAs). This schematic representation illustrates the reported patterns of cross‐reactivity among different GBCAs in patients with immediate (IgE‐mediated) and non‐immediate (T‐cell‐mediated) hypersensitivity reactions. The agents are grouped according to their structural class (linear vs. macrocyclic) and ionicity (ionic vs. non‐ionic). Red arrows indicate potential cross‐reactivity between agents, as documented in clinical reports, skin testing, or drug provocation testing. The direction of the arrows reflects the clinical sequence or substitution patterns observed in the literature. However, it should be noted that the strength and reproducibility of these cross‐reactivity pathways vary, and the underlying mechanisms (e.g., shared excipients, molecular similarity) remain incompletely understood. This figure aims to support clinicians in the risk stratification and selection of alternative GBCAs in patients with a documented hypersensitivity reaction. CR, cross‐reactivity; DPT, drug provocation test; GBCA, gadolinium‐based contrast agent; ST, skin test. Created in BioRender.

**FIGURE 3:**
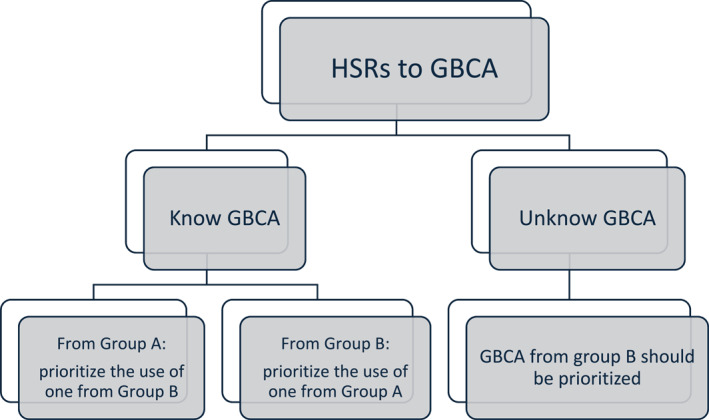
The empirical selection of an alternative GBCA in case of previous HRs (adapted from Vega et al. [14]).

CR among GBCAs is still unclear, although reported. Most studies investigating CR demonstrate poor methodologic quality: small sample size, retrospective analysis, different research protocol, a battery of GBCAs skin tests, and drug provocation tests (DPTs) are not always performed. CR was studied, especially for IHSRs and GBCAs, but the reported data are extremely scarce. No data about CR in NIHSRs is available. Grueber et al. observed CR in one‐third of the patients (about 29%) with IHSRs [43]. Patterns of CR have been reported above all within the macrocyclic group where monosensitization to gadobutrol [41] or gadoteridol [34] may occur between macrocyclics and linears; apparently, linears do not cross‐react with each other, although in one case of CR has been described [38, 41]. Mankouri et al. calculate a CR rate of 27.7% among macrocyclic agents, 5.5% among linear agents, and 5.5% between both [38]. In some countries, including the European Union (EU), the use of linear GBCAs has been restricted or suspended by medical authorities because of their risk of nephrogenic systemic fibrosis and gadolinium deposition in the brain [4], with difficulty in searching for an alternative GBCA in case of HRs to macrocyclic GBCAs. Ryoo et al. found, in patients with a history of mild IHSRs, a recurrence rate of up to 26% reusing the culprit GBCA, up to 12% given a different GBCA with the same molecular structure, and only 6% changing the GBCA with a distinct molecular structure class [44]. It would be important to clarify the CR pattern better to select a safe alternative [6]. According to the studies, STs with GBCAs show a high NPV at 86%–89% [34, 36], up to 100% [34]. Therefore, they are essential to find a substitute GBCA even if the DPT remains mandatory because a positive drug challenge may occur despite negative STs [45]. Recently, Vega et al. have proposed an empirical approach to choosing an alternative GBCA without a formal allergy work‐up to assess the pattern of CR among GBCAs [14]. Similarly to the already known classification of ICM according to the side chain, the authors classify the macrocyclic GBCAs available in the EU into three homogeneous groups (A, B, C) according to the chemical structure of the chelating agent (Figure 1). Potential patterns of CR of GBCAs are shown in Figure 2. The empirical selection of another GBCA in the case of previous HRs is explained in Figure 3. This should only be used if an allergy evaluation cannot be performed.

### 3.5 The Drug Provocation Test (DPT) in GBCAs HSRs

DPT is the gold standard for diagnosing HSRs to drugs [14]. This also applies to HSRs and GBCAs, although there are no standardized procedures regarding contrast media allergy [46]. DPT is poorly studied and there is no consensus on the protocol that should be used. A recent review by Gracia Bara MT et al. analyzed some studies in which DPT was performed when skin tests were negative [1]. The review found no consensus on total dose and administration intervals. The doses ranged from 1 to 10 mL. The protocol used for DPTs is rarely specified, as shown in Table 1, probably because most were case reports or case series with a limited number of patients. The absence of consensus concerns both IHSRs and NIHSRs. In the study by Seta V. et al., a patient with a nonimmediate hypersensitivity reaction was exposed to a single‐blind placebo‐controlled DPT with 1 mL of GBCA [36]. In a recent work by Stehlin, involving patients with a history of IHSRs to GBCAs, the protocol provided intravenous placebo administration with 5 mL of 0.9% NaCl followed by a 2‐step challenge with 1 and 4 mL separated by a 30‐min interval. For a 50 kg patient, this dosage corresponds to a 100% dose of gadobutrol and a 50% dose of gadoteridol and gadobenate [35].

**TABLE 1:** State of art of DPTs in GBCAs HSRs.

Study	Patients	Timing of reaction	Positive ST	Tolerated GBCAs	Non tolerated GBCAs	Protocols
Moulin et al. [47]	1	IHR	Gadoterate meglumine	Gadobenate dimeglumine	—	Unspecified/re‐exposed
Chiriac et al. [48]	27	IHR	Gadobenate dimeglumine (in 1 patient)	Gadopentetate dimeglumine Gadobenate dimeglumine Gadoterate meglumine Gadoteridol	—	Unspecified/re‐exposed
Kolenda et al. [41]	33	IHR	Gadobenate dimeglumine Gadoterate meglumine Gadobutrol (in 19 patients, 57%)	Gadobenate dimeglumine Gadoterate meglumine Gadobutrol	—	Unspecified/re‐exposed
Moreno‐Escobosa et al. [45]	1	IHR	Gadobenate dimeglumine Gadodiamide Gadoterate meglumine	—	Gadoteridol	Unspecified/re‐exposed
Seta et al. [36]	14	IHR and NIHR	Gadobutrol Gadoteridol Gadoterate meglumine (all positive) NPV 86%	Gadoterate meglumine Gadopentetatedimeglumine	Gadoterate meglumine Gadoteric acid	Single‐blind placebo‐controlled DPT, during 8‐hours hospital stay with 1 mL of the GBCA (1/10 of usually injected dose for MRI)
Hojreh et al. [49]	17	IHR	—	Gadobenate dimeglumine Gadoterate meglumine Gadobutrol	—	Unspecified/re‐exposed
Mankouri et al. [38]	132	IHR and NIHR	Gadobenate dimeglumine gadoterate meglumine gadobutrol gadoteridol (in 18 patients, 13,6%)	Gadodiamide Gadoterate meglumine Gadobutrol	—	Unspecified/re‐exposed
Gallardo et al. [50]	5	IHR	Gadoterate meglumine Gadobutrol (in 2 patients)	Gadoxetate disodium Gadoterate meglumine	Gadobutrol	Unspecified/re‐exposed
Vega et al. [34]	16	INH and NIHR	Gadobutrol (in 1 patient)	Gadoteric acid (0.2 mg/kg)	Gadoteric acid Gadobutrol (0.1 mg/kg)	1/3 dose at a rate of 120 cc/h and, immediately after, the remaining 2/3 at 40 cc/h; total infusional time 8′
Stehlin et al. [35]	9	IHR	Gadobenate Gadobutrol gadoteridol (in 3 patients)	Gadobenate Gadobutrol gadoteridol	Gadobenate	2‐Step, placebo‐controlled DPT, 1 mL IV (1/5 dose), if negative after 20‐30′ 4 mL IV, then evaluation after 60′
Macias et al. [26]	1	NIHR	Gadoxetate disodium	—	Gadoteric acid	
Rodriguez‐Nava et al. [36]	1	IHR	Gadobenate dimeglumine gadoterate meglumine	—	Gadobutrol	10% dose; monitor for 30 min followed by rest of the dose and monitored for 1 h
Gallardo et al. [51]	1	NIHR	—	Gadoxetate disodium (1^st^ day 453.58 mg, 2^nd^ day 1360.73 mg)	Gadobutrol (1^st^ day 1209.44 mg, 2^nd^ day 4535.4 mg)	2‐day, with 1 week of delay

*Note:* We highlighted, when available, the positivity of skin tests, the tolerated GBCAs, and the protocols for DPTs. Unfortunately, the lack of data on sensitivity, specificity, and predictive values is a major limitation of the papers taken into account, which were mostly case reports and case series.

The tested dose represents a very important aspect. Dosing 1 mL (for gadoteric acid represents 1/10th of the total dose) could carry the risk of missing dose‐dependent non‐IgE mediated reactions. On the other hand, a full dose exposes the patient to the risk of GBCAs toxicity. Attention should be paid to the possible AEs caused by contrast agents, although they are well‐tolerated agents. GBCAs are nephrotoxic and can be deposited in different organs [52]. Therefore, it is necessary to have precautions such as those taken in radiology, asking about the possible presence of renal disease, the last performance of contrastography investigations, or the intake of nephrotoxic drugs. Vega et al. proposed a fast challenge test. They highlighted another critical point: GBCAs in clinical practice are given in bolus, so graded challenges could cause false negative results [34].

Regarding NIHSRs, Gallardo et al. reported a case of maculopapular exanthema in a 49‐year‐old woman 24 h after a cardiac NMR with gadobutrol. A complet. allergy evaluation was carried out one month after the NIHSR, using patch tests (PTs) and STs with a wide panel of GBCAs; both resulted in negative at 6‐, 48‐ and 96‐h readings. DPTs in 2 days with the culprit were performed due to mild skin reaction and the test results, but 15 h after the DPT, an itchy erythematous rash appeared. Later, the patient tolerated a 2‐day DPT with alternative GBCAs, particularly gadoxetate disodium. Notably, the latter belong to a linear group of GBCAs [51]. A history of SCARs contraindicates DTPs with the suspect drug, despite negative STs, and it is preferred to avoid DPTs also with structurally related drugs due to the risk of a reaction relapse. The pediatric case report from Macías‐Iglesias et al., cited above, showed a recurrence of DRESS after a DTPs with alternative GBCAs, despite negative STs [26].

### 3.6 Approach to Premedication

Initially introduced to reduce the IHSRs to high osmolality ICM, corticosteroid premedication regimens have been extended to GBCAs. Although commonly performed in clinical practice, the efficacy of premedication before repeat GBCA exposure in patients with a history of hypersensitivity reactions is a controversial topic. Different studies have reported the ineffectiveness of premedication as prophylaxis of GBCA‐associated hypersensitivity reactions [47] with breakthrough responses in up to 39% of cases using the same GBCA despite premedication [48]. In contrast, Ahn et al. suggested a preventive effect of premedication for lowering the recurrence rate of immediate and delayed hypersensitivity reactions [21].

Usually, the premedication protocol consists of a combination of a multidose corticosteroid and an antihistamine. A 12‐h [53] or 13‐h [54] oral scheme of steroids is usually recommended based on the pharmacokinetics of corticosteroids [55]. However, a rapid premedication regimen (e.g., hydrocortisone, 200 mg intravenously, immediately and every 4 h until the procedure is completed and diphenhydramine, 50 mg intravenously, 1 h before the procedure) is recommended in emergency settings [56].

Premedication in primary prevention is considered unnecessary. However, like ICM, varying approaches have been proposed for patients who have experienced HSRs, although the studies evaluating alternative strategies are few and underpowered. An empirical switch with alternative GBCA, with or without premedication, has been explored in a single‐center study, observing a substantial reduction in the hypersensitivity reaction rate [50]. Evaluation for GBCAs HSRs via STs to identify subgroups with an underlying allergy is being performed. Although the sensitivity of skin tests is suboptimal, the NPV appears to be high, and the extent of CR among GBCAs is still unclear; therefore, STs are pivotal in selecting alternative GBCAs [6].

Lastly, owing to the paucity of immediate hypersensitivity reaction rates after nonvascular administration, the ESUR no longer recommends the concomitant administration of systemic corticosteroids and antihistamines before the nonvascular infusion of contrast media [58, 59].

Usually, studies put the benefit of premedication at the center of attention. Still, corticosteroids also carry direct and indirect risks, especially in certain groups of patients or when premedication is no longer useful. Direct risks such as transient leukocytosis, hyperglycemia, or increased infection should be considered. Indirect risks regarding the inpatient population are more difficult to estimate and are represented by prolonged hospital stays and increased rates of hospital‐acquired infections [59]. Nowadays, there is no consensus or discrepancies between international societies regarding premedication.

## 4 Conclusions

The global use of GBCAs has increased significantly in recent years. While these agents are generally considered safe, adverse events, including HSRs, can still occur. Although infrequent, HSRs may be severe and require careful management. In all suspected cases, a comprehensive allergological assessment is essential to confirm the diagnosis and to identify a well‐tolerated alternative agent when future imaging is needed.

One of the most debated issues is the role of premedication. Traditionally used in patients with a history of immediate reactions to contrast media, recent evidence suggests its effectiveness is limited, particularly in preventing recurrence. Antihistamines may help mask early symptoms of anaphylaxis, but they do not offer protection against more serious outcomes. Corticosteroids, while frequently used, may expose patients to unnecessary side effects and should be considered with caution [22, 47, 49, 51, 52, 55, 60, 61].

Furthermore, current diagnostic tools such as STs and DPT play a central role in the work‐up of suspected GBCA allergy. However, the available evidence remains limited, often based on case reports or small retrospective studies. The cross‐reactivity patterns among different GBCAs are not yet fully defined, and more robust, prospective studies are needed to optimize diagnostic and therapeutic pathways [1, 6, 14, 38].

Improving communication and collaboration between radiologists and allergists is essential. Shared decision‐making and multidisciplinary protocols will help ensure safer diagnostic procedures for patients with a suspected or confirmed allergy to GBCAs.

To summarize, here are the main practical points for clinical application:

- A detailed clinical history is the cornerstone of evaluating suspected GBCA hypersensitivity.
- STs offer a high negative predictive value and may help identify safe alternatives, but DPT should complement them when feasible.
- Premedication is not universally protective and should not replace proper diagnostic evaluation; its use must be individualized.
- Preference should be given to macrocyclic GBCAs, which are associated with lower tissue retention and may reduce the risk of HSRs.

Establishing a structured collaboration between radiology and allergy teams is crucial for accurate diagnosis, patient safety, and optimal imaging strategies.

## Author Contributions

Conceptualization, F.L. and G.P.; writing – original draft preparation, F.L., G.P., G.C., F.L.R., M.M., D.P., F.R., A.S., A.T., A.V.; writing – review and editing, F.L., G.P., M.T.C., G.C., F.L.R., M.M., D.P., F.R., A.S., S.T., A.T., A.V.; supervision, M.T.C., E.H., G.W.C., M.D.G. All authors have read and agreed to the published version of the manuscript.

## Conflicts of Interest

Giovanni Paoletti reports fees for speaker activities and/or advisory boards participation from Lofarma, GSK, and AstraZeneca, outside the submitted work. Giorgio Walter Canonica reports research or clinical trials grants paid to his Institution from Menarini, AstraZeneca,GSK, Sanofi Genzyme and fees for lectures or advisory board participation from Menarini, AstraZeneca, CellTrion, Chiesi, Faes Farma, Firma, Genentech, Guidotti‐Malesci, GSK, HAL Allergy, Innovacaremd, Novartis, OM‐Pharma, Red Maple, Sanofi‐Aventis, Sanofi‐Genzyme, Stallergenes‐Greer and Uriach Pharma, outside the submitted work. Enrico Heffler reports fees for speaker activities and/or advisory boards participation from Sanofi, Regeneron, GSK, Novartis, AstraZeneca, Stallergenes‐Greer, Chiesi, Almirall, Bosch, Lofarma, outside the submitted work. The other authors declare no conflicts of interest in this paper.

## Data Availability

Data sharing not applicable to this article as no datasets were generated or analyzed during the current study.
